# Schistosomiasis, intestinal helminthiasis and nutritional status among preschool-aged children in sub-urban communities of Abeokuta, Southwest, Nigeria

**DOI:** 10.1186/s13104-017-2973-2

**Published:** 2017-11-28

**Authors:** Adebiyi Abdulhakeem Adeniran, Hammed Oladeji Mogaji, Adeyinka A. Aladesida, Ibiyemi O. Olayiwola, Akinola Stephen Oluwole, Eniola Michael Abe, Dorcas B. Olabinke, Oladimeji Michael Alabi, Uwem Friday Ekpo

**Affiliations:** 1 Department of Pure and Applied Zoology, Federal University of Agriculture, Abeokuta, Nigeria; 2 Department of Nutrition and Dietetics, Federal University of Agriculture, Abeokuta, Nigeria; 3 Department of Zoology, Federal University Lafia, Lafia, Nigeria

**Keywords:** Preschool aged children, STH, Schistosomiasis, NTDs control, Malnutrition

## Abstract

**Objective:**

Schistosomiasis and intestinal helminthiasis are major public health problems with school-aged children considered the most at-risk group. Pre-school aged children (PSAC) are excluded from existing control programs because of limited evidence of infections burden among the group. We assessed the prevalence of infections and effect on nutritional status of preschool aged children in Abeokuta, Southwestern Nigeria.

**Results:**

A community-based cross-sectional study involving 241 children aged 0–71 months was conducted in 4 sub-urban communities of Abeokuta. Urine and faecal samples were collected for laboratory diagnosis for parasites ova. Nutritional status determined using age and anthropometric parameters was computed based on World Health Organization 2006 growth standards. Data were subjected to descriptive statistics analysis, Chi square, t-test and ANOVA. Of 167 children with complete data, 8 (4.8%) were infected with *Schistosoma haematobium*; *Schistosoma mansoni* 6 (3.6%); *Taenia* species 84 (50.3%); *Ascaris lumbricoides* 81 (48.5%) and hookworm 63 (37.7%). Overall, 46.7% of the children were malnourished, 39.5% stunted, 22.8% underweight and 11.4% exhibiting wasting/thinness. Mean values of anthropometric indices were generally lower in children with co-infection than those with single infection. We observed low level of schistosomiasis but high prevalence of intestinal helminthiasis and poor nutritional status that calls for inclusion of PSAC in control programs.

**Electronic supplementary material:**

The online version of this article (10.1186/s13104-017-2973-2) contains supplementary material, which is available to authorized users.

## Introduction

Nigeria is the most endemic Sub-Saharan African country for schistosomiasis and intestinal helminthiasis [1–4]. These diseases are closely linked to poverty, lack or inadequate safe water, sanitation and hygiene [5–8]. School-age children (SAC), pregnant women and PSAC are particularly at risk of morbidity of infection [9]; with chronic infection compromising growth, development, cognition, iron status and naivety of immune system which further increase susceptibility to infections [10].

Blood losses from haematuria and faecal occult blood from schistosomiasis affects iron balance and subsequently nutrition [11]. Intestinal helminths given their peculiar niche also deprive hosts of essential nutrients. And endemicity in less-developed country like Nigeria is high such that the gastrointestinal tract of a child is often parasitized with at least one of the three most occurring geohelminths including *A. lumbricoides, T. trichiura* and hookworms [9, 10]. In fact, PSAC comprise between 10 and 20% of the 3.5 billion people living in soil transmitted helminths (STH) endemic areas [10, 12]. PSAC are nutritionally vulnerable segment of the population and defects during this developmental stage may persist for long and sometimes throughout life [13]. Nutritional status of infected individual is altered through decline food intake, increase in nutrient wastage through loss of blood, vomiting, diarrhoea and can be aggravated by helminths infection [14, 15].

Dearth of information on burden of *Schistosoma* spp. and intestinal helminths infections in PSAC relative to SAC has been given as reason for exclusion from existing control programs [3, 10]. We investigate the prevalence of schistosomiasis, intestinal helminthiasis and nutritional status among PSAC living in sub-urban communities in Abeokuta, Nigeria.

## Main text

### Methods

A community-based cross-sectional survey was carried out in Ago-Ika, Ikereku-Idan, Itun-Seriki and Adebowale-Abowaba, communities in Abeokuta, Ogun State (Additional file 1: Figure S1) from February to June 2014. The communities are sub-urban without internal road network, with traditional mud houses and toilet facilities outside of the houses at considerable distance. There were no efficient drainage facilities, and predominant occupations include trading, artisans with few civil workers.

As no consistent definition of PSAC exists in medical literature in terms of age limit and/or school enrolment [10], PSAC for this study was defined as children aged 0–5 years; inclusive of children aged 5 years but yet to reach 6th birthdate irrespective of school enrolment status [3, 16]. Sample size was calculated from a total population figure of 2877 for children aged 0–5 years (ANLG Primary Health Care Unit, 2013). Using the method of [17] and estimated prevalence of 17% [18], a final sample size of 220 was calculated. Exhaustive sampling was done to ensure calculated sampling size was met with all households within each of the community with PSAC visited.

Ethical clearance was obtained from the Ethics review committee of the Federal Medical Centre, Abeokuta with Reference Number FMCA/238/HREC/14/2013. Approval to conduct the study was equally obtained from the Ogun State Ministry of Health and enrolment was after informed consent were obtained from parents/caregivers.

Pre-tested questionnaires (Additional file 2) developed were interviewer-administered to consented participants to obtain demographic data including age, gender and investigate knowledge; attitude and practices (KAP) exposing PSAC to infection with the assistance of parents/caregivers. Sample collection and handling was as previously described [18–20]. To explain briefly, two labelled, sterile universal plastic bottles were given to parents/caregiver for collection of midday urine (10.00 a.m.–1.00 p.m.) and faecal samples. Urine, collected in dark containers to prevent hatching of ova on exposure to sunlight, and faecal samples were then taken to the laboratory for analysis.

Anthropometric data of height, recumbent length (children < 2 years) and weight were obtained as described [19, 21]. Urine samples were examined for micro-haematuria and *S. haematobium* ova using reagent strip and sedimentation method [20]. Faecal samples were processed using sodium-acetate acetic-acid formalin ether concentration method and examined for parasites ova under the microscope [22].

Data were analysed using IBM SPSS 20.0 (IBM, NY). Descriptive statistics was used to describe categorical variables. Chi square, t-test and ANOVA were used to test for significance. Nutritional status was determined using anthropometric height-for-age (HAZ), weight-for-age (WAZ), weight-for-height (WHZ), and body-mass-index-for-age (BAZ) z-scores calculated and compared with WHO 2006 child growth standard. Significant level was set at p < 0.05.

## Results

A total of 241 PSAC were enrolled for the study with 167 (69%) providing urine, faecal samples and complete questionnaire data (Additional file 3: Figure S2). Of these, 83 (49.7%) were males, and 84 (50.3%) were females with mean age of 3 years (Additional file 4).

Eleven (6.6%) PSAC had micro-haematuria with 6 (3.6%) children positive for *S. haematobium* infection. Females had higher mean intensity of infection (0.464/10 ml of urine) than the males (0.151/10 ml of urine) (p = 0.178). Prevalence of *S. mansoni* was 8 (4.8%) with males having higher mean egg per gram of faeces (0.259 epg) than the females (0.181) with no significant difference (p = 0.693). Intensity of infection increases with age in both *Schistosoma* infections. Six intestinal helminths including *Taenia* spp. (50.3%), *A. lumbricoides* (48.5%), hookworm (37.7%), *T. trichiura* (5.4%), *Strongyloides stercoralis* (4.2%) and *Trichostrongylus* spp. (4.2%) were observed with no significant difference by age and sex except infection by age in *T. trichiura* and *Taenia* spp. (p = 0.025 and p = 0.000 respectively). *Taenia* spp intensity was significantly different (p = 0.046) across age group (Table 1). Prevalence of co-infection with schistosomiasis and intestinal helminths was 6.0% with females 7 (70%) and 0–24 months 6 (60%) more infected.

**Table 1:** Prevalence and intensity of parasitic infection by sex and age group

	*S. haematobium*		*S. mansoni*		*A. lumbricoides*		*Hookworm*		*T. trichiura*		*Taenia* spp.		*S. stercoralis*		*Trichostrongylus* spp.	
	NI (%)	GMI^a^	NI (%)	GMI (epg)	NI (%)	GMI (epg)	NI (%)	GMI (epg)	NI (%)	GMI (epg)	NI (%)	GMI (epg)	NI (%)	GMI (epg)	NI (%)	GMI (epg)
Sex																
Male	2 (33.3)	0.151	3 (37.5)	0.259	40 (49.4)	1.66	31 (49.2)	1.28	2 (22.2)	0.000	40 (47.6)	1.01	4 (57.1)	0.977	3 (42.9)	0.78
Female	4 (66.7)	0.464	5 (62.5)	0.181	41 (50.6)	1.64	32 (50.8)	1.44	7 (77.8)	0.506	44 (52.4)	1.22	3 (42.9)	0.401	4 (57.1)	0.37
p-value	0.414	0.178	0.479	0.693	0.936	0.925	0.921	0.368	0.087	0.217	0.588	0.107	0.687	0.074	0.711	0.450
Age (months)																
0–12	2 (33.3)	0.151	3 (37.5)	0.201	12 (14.8)	1.51	7 (11.1)	1.29	–	–	4 (4.8)	1.00	1 (14.3)	0.301	–	–
12–24	2 (33.3)	0.301	2 (25.0)	0.000	14 (17.3)	1.38	12 (19.0)	1.33	1 (11.1)	0.301	17 (20.2)	0.87	–	–	1 (14.3)	0.78
25–36	–	–	1 (12.5)	0.477	16 (19.8)	1.89	10 (15.9)	1.31	5 (55.6)	0.49	17 (20.2)	1.01	2 (28.6)	0.57	1 (14.3)	0.00
36–48	2 (33.3)	0.628	1 (12.5)	0.602	11 (13.6)	1.70	11 (17.5)	1.22	–	–	13 (15.5)	1.19	1 (14.3)	0.60	2 (28.6)	0.15
48–60	–	–	–	–	12 (14.8)	2.16	10 (15.9)	1.54	1 (11.1)	0.78	15 (17.9)	1.53	1 (14.3)	0.60	–	–
61–71	–	–	1 (12.5)	0.000	16 (19.8)	1.33	13 (20.6)	1.47	2 (22.2)	0.00	18 (21.4)	1.10	2 (28.6)	1.23	3 (42.9)	0.91
Total	6 (3.6)	0.360	8 (4.8)	0.210	81 (48.5)	1.65	63 (37.7)	1.36	9 (5.4)	0.49	84 (50.3)	1.12	7 (4.2)	0.73	7 (4.2)	0.54
p-value	0.324	0.145	0.702	0.125	0.508	0.182	0.370	0.917	0.025	0.634	0.000	0.046	0.725	0.54	0.270	0.58

*NI* number infected, *GMI* geometric mean intensity of infection

^a^egg/10 ml of urine

Out of the total population, 78 (46.7%) were malnourished with no significant difference between sexes (p > 0.05). Approximately 23% were underweight (WAZ < − 2SD), 39.5% stunted (HAZ < − 2SD) and 11.4% wasting (WHZ < − 2SD)/thin (BAZ < − 2SD). Also, 28 (73.7%) of underweight, 51 (77.3%) of stunted and 14 (73.7%) of wasting/thin were infected with intestinal helminths respectively (Table 2). However, there was no significant difference between infected and non-infected PSAC. Also, 5.3, 4.5 and 5.9% of PSAC underweight, stunted and wasted were infected with *Schistosoma* spp. respectively. Mean Z-scores of nutritional indicators were generally lower in infected than non-infected children but not significantly different (Additional file 4).

**Table 2:** Nutritional indicator and parasitic infection

	Nutritional indicator		
	Underweight (WAZ < − 2SD)	Stunting (HAZ < − 2SD)	Wasting/thinness (WHZ < − 2SD)/(BAZ < − 2SD)
	NI (%)	NI (%)	NI (%)
Intestinal helminths infection			
Positive	28 (73.7)	51 (77.3)	14 (73.7)
Negative	10 (26.3)	15 (22.7)	5 (26.3)
p-value	0.551	0.995	0.694
Schistosomiasis			
Positive	2 (5.3)	3 (4.5)	1 (5.3)
Negative	36 (94.7)	63 (95.5)	18 (94.7)
p-value	0.602	0.286	0.730
Co-infection of schistosomiasis and intestinal helminths			
Positive	2 (5.3)	3 (4.5)	0 (0)
Negative	36 (94.7)	63 (95.5)	19 (100)
p-value	0.830	0.525	–
Total	38 (22.8)	66 (39.5)	19 (11.4)

Approximately 66% of the parents interviewed have no knowledge of schistosomiasis or its mode of transmission, and 61% of the studied PSAC had been exposed to the river with more than half before their first birthday (Table 3). Bathing (20.2%) is the major activity predisposing PSAC to infection, and 63.6% were bathed with water fetched from the river at home.

**Table 3:** Knowledge, attitude and practices and water, sanitary and hygiene practices of parents that predispose PSAC to infection

KAP of parents	Number (%)
Knowledge about schistosomiasis (N = 104)	
Yes	35 (33.7)
Exposure of PSAC to Ogun River (N = 167)	
Yes	103 (61.7)
Age of first exposure (N = 103)	
At birth	15 (14.5)
Before first year	52 (50.5)
2–5 years	36 (35.0)
Major activity exposing PSAC to infection source (N = 104)	
Bathing	21 (20.2)
Washing	9 (8.7)
Fetching	5 (4.8)
Recreational	4 (3.8)
Bathing and washing	10 (9.6)
Washing and fetching	2 (1.9)
Others	4 (3.8)
Means of exposure to water from river (N = 66)^a^	
Child was taken to the river	20 (30.3)
Water from the river used to bathe child at home	42 (63.6)
Child goes to river by himself	4 (6.1)
Hand washing practice before feeding PSAC (NE = 41)^b^	
Yes	37 (90.2)
Frequency of deworming preschool-aged children (N = 104)	
Always	42 (40.4)
Rarely	32 (30.8)
Never	30 (28.8)
Do you clean your child hand after defaecation?	
Yes, with water	52 (50.0)
Yes, with water and soap	33 (31.7)

Water, sanitary and hygiene practices of PSAC	Number (%)
Type of toilet facility used	
Water closet	34 (20.4)
Pit with slab	81 (48.5)
Open pit latrine	22 (13.2)
Bush	27 (16.2)
River	3 (1.8)
Main water source for domestic use	
Tap	83 (49.7)
River	22 (13.2)
Well	5 (3.0)
Multiple source	57 (34.1)
PSAC picking food/objects from the ground	
Yes	53 (31.7)
Hand washing practices of PSAC before eating	
Yes, with water	70 (41.9)
Yes, with water and soap	15 (9.0)
No, not applicable	82 (49.1)
Preschool-aged children with dirty finger (NE = 167)	
Dirty	106 (63.5)
Clean	61 (36.5)
PSAC with trimmed fingernails (NE = 167)	
Trimmed	99 (59.3)
Not trimmed	68 (40.7)
PSAC that had slippers/shoes (NE = 104)	
Yes	96 (92.3)
Frequency of wearing slippers/shoes in PSAC	
Always	58 (55.8)
Seldom	38 (36.5)
Don’t wear	8 (7.7)

*PSAC* Pre-school aged children

^a^Question answered by parents of preschool-aged children that were exposed to infection source

^b^Parents of breastfeeding preschool-aged children

Indiscriminate defaecation is widespread in the study communities as 16.2% of the parent/caregiver engaged in open defaecation in surrounding bushes and 1.8% directly to the river. Although, 49% of household uses public tap water for domestic purposes, 13.2% still depends solely on water from the river for domestic usage. Only 9% of PSAC that have started consumption of staple foods have their hands washed with water and soap before eating. Of all the PSAC studied, 63.5% have dirty fingers and only 59.3% have their fingernails trimmed. 92% of PSAC have slippers/shoe, and 55% wear their shoes always (Table 3).

## Discussion

Schistosomiasis and intestinal helminths are major public health problems in sub-Saharan Africa with Nigeria being the most endemic for both diseases [1]. Inclusion of PSAC in MDA treatment campaigns with anthelmintic, the major control intervention in Nigeria is necessary to actualize WHO vision to control/eliminate infections in endemic countries by 2020 [3, 10]. However, relative to overwhelming evidences of infection burden in SAC; available information in PSAC is less and further evidences are required. This study provides the first report of *S. mansoni* infection in PSAC in Abeokuta. Though observed prevalence is low, this is a drawback on control efforts as current estimate might not be reflective of actual infection burden. Emergence of genitourinary schistosomiasis in PSAC could be linked to poor sanitary behaviours and unsafe water contact practices in which children were exposed to infection source before their first birthday. Mothers/caregivers inadvertently expose their wards to *Schistosoma* infection when bathing them with infected water or when these children accompany them to the river for domestic activities [3]. Educating mothers and caregivers on transmission routes and the role they play in exposing children to infection is thus critical to the success of control program.

High prevalence of intestinal helminthiasis observed provides evidence for the need for inclusion of PSAC in control programme using available opportunities [10]. Prevalence of *A. lumbricoides* infection was higher than other reports in the country [23, 24] and compares with infection in SAC [25]. However, prevalence of hookworm and *T. trichiura* is lower [10, 26–29]. Loss of essential nutrients necessary for healthy development, impairments in physical, intellectual and cognitive development are some of the burden of infection on PSAC and should be of public health concern [10]. *A. lumbricoides* infection could result from contamination of household utensils, food and drinking-water handling equipment with parasite’s ova if safe human waste disposal methods or hand washing facilities are lacking [25]. Hygiene practices were poor among the study population. Even though respondents claim regular hand-washing practice before eating, it is common for children to share foods when playing and they did so without washing hands. Also, majority of the PSAC reported possession and regular wearing of footwears, the veracity of these responses is unverifiable due to unwillingness of respondents to disclose their social status. Furthermore, children wearing protective shoes often remove it when playing on soil because they mostly have one and need to make the shoe last longer; thereby getting exposed to active penetration of infective hookworm larvae when playing in contaminated soil in their environment [25]. Inadequate use of footwears is a risk factor in the transmission of hookworm infections [19, 27].

The prevalence of *Taenia* spp. observed in the study is higher than previous report [30]. The high occurrence of *Taenia* spp. in PSAC requires further investigation to ascertain the route of transmission. The infection may have been acquired from consumption of improperly cooked meat/pork as it is common for parents/caregivers to feed PSAC with undercooked adult meals like fish, meat and beans aimed at hastening transition from breastfeeding to staple foods. Improper human faecal disposal predominant in the study communities is one of the factors responsible for sustaining transmission of *Taenia* spp. which may be acquired at any age from 2 years onward [30, 31]. This reinforces the need for inclusion of PSAC in chemotherapy as praziquantel administration is suitable for the treatment of both schistosomiasis and *Taenia* spp. infection.

Although probability of co-infection is high, no report exists for co-infection of schistosomiasis and intestinal helminths in PSAC in Nigeria. The prevalence of co-infection and exposure pattern to risk factors observed is similar to reports in SAC [3, 32]. Similarity in infection level and continual exclusion from preventive chemotherapy could be a major reason why PSAC constitute an important reservoir of helminths infection [33].

There was no significant relationship between the high prevalence of malnutrition and parasites infection, which may be due to low infection intensity. Similar observation has been reported among PSAC infected with geohelminths [19]. Correlation analysis, although not significant, showed negative associations between helminths and nutritional status (Additional file 4); implying that a threshold could exists with which parasitic infection will have significant effect on nutritional status when other factors affecting nutrition are controlled. Age could also be a limiting factor given that the effects of these diseases are more pronounced in the long-term and might not be easily measured in the early developmental stage of PSAC. Causes of malnutrition are multifactorial and the social, economic and physical environment in which individual live are major determinant of the degree of association between parasites and nutritional status [34]. Nevertheless, we found evidence that infection of schistosomiasis and intestinal helminths contributes a portion of risk to the development of malnutrition in PSAC and are as much of a public health problem as in SAC. The long-term effects of infection on growth, development and cognition of PSAC are enormous and cannot be ignored, even though assessing nutritional impacts of endemic infections could be quite difficult [35]. These factors are further complicated by high prevalence of malnutrition and if not dealt with earlier, may contribute to reduced school attendance and higher susceptibility to disease, thereby compromising physical capacity and work opportunities in adulthood. As no intervention exists for helminths control in PSAC in Nigeria, this study in accord with WHO recommendation calls for inclusion of PSAC in helminths interventions in Nigeria.

### Limitation of the study

The sample size limits the power of our findings and calls for caution in generalization of study findings. This was majorly due to political tension created during the study period that made considerable number of participants withdrawn (Additional file 2).

## Additional files

**Additional file 1: Figure S1.** Map of study area with study locations.

**Additional file 2.** Questionnaire used for the study.

**Additional file 3: Figure S2.** Flowchart of the study.

**Additional file 4.** Demographics, means Z-score of nutritional indicators used, associated risk factor of infection and correlation analysis.

## Abbreviations

PSAC: pre-school aged children
SAC: school aged children
STH: soil transmitted helminths
WHO: World Health Organisation
ANLG: Abeokuta North Local Government
MDA: Mass Drug Administration
