# Supplementary material for: Schistosomiasis, intestinal helminthiasis and nutritional status among preschool-aged children in sub-urban communities of Abeokuta, Southwest, Nigeria
Source: BMC Res Notes. 2017 Nov 28;10:637. doi: 10.1186/s13104-017-2973-2 (PMC5706406; doi:10.1186/s13104-017-2973-2)
Supplement: Supplementary file 1 — Additional file 1: Figure S1. Map of study area with study locations. [file 13104_2017_2973_MOESM1_ESM.docx]

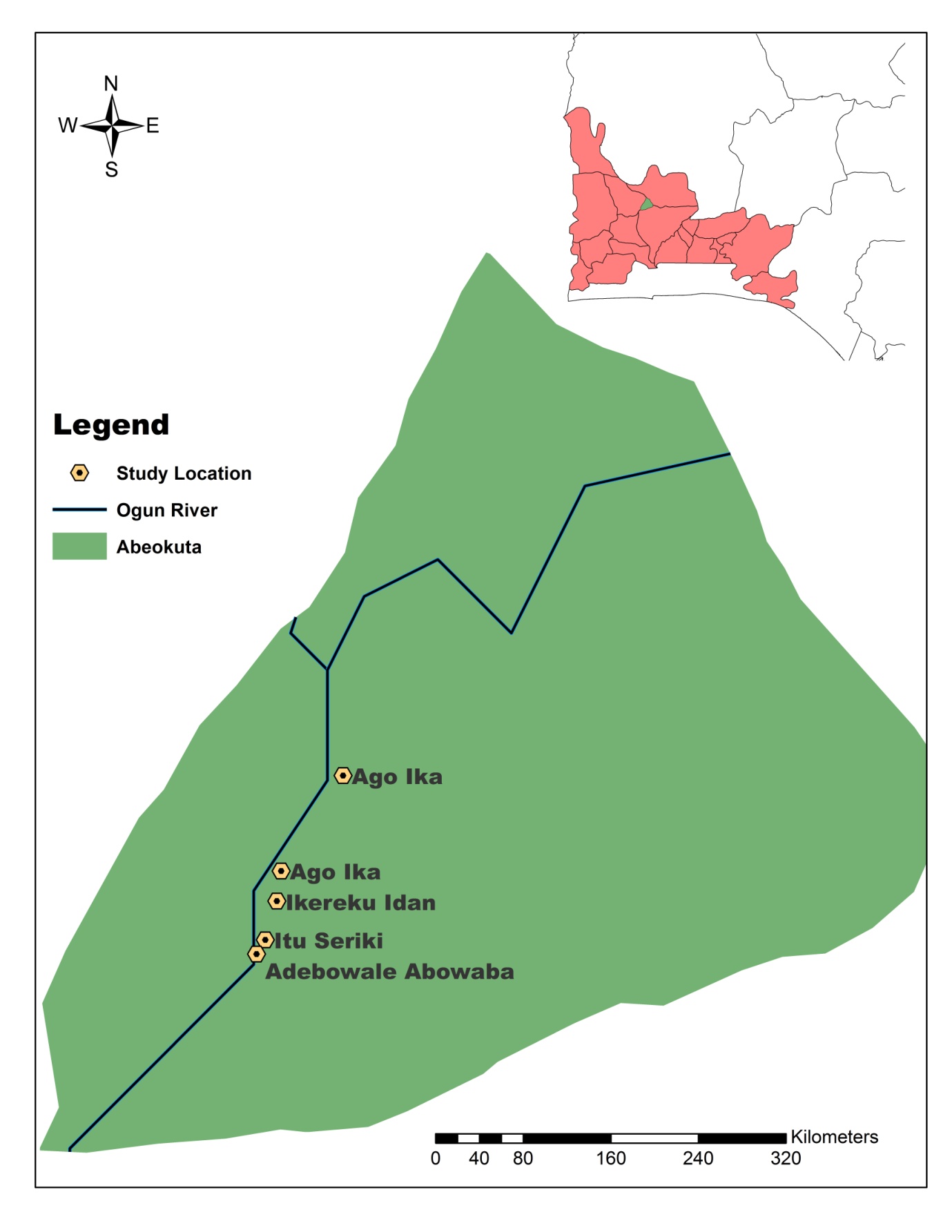


Figure S1A: Map of study area showing the study locations (Source: Map was created using ESRI ArcGIS® 9.3 software)
