# Supplementary material for: Schistosomiasis, intestinal helminthiasis and nutritional status among preschool-aged children in sub-urban communities of Abeokuta, Southwest, Nigeria
Source: BMC Res Notes. 2017 Nov 28;10:637. doi: 10.1186/s13104-017-2973-2 (PMC5706406; doi:10.1186/s13104-017-2973-2)
Supplement: Supplementary file 2 — Additional file 2. Questionnaire used for the study. [file 13104_2017_2973_MOESM2_ESM.docx]

**QUESTIONNAIRE FOR RESEARCH ON: Schistosomiasis, intestinal helminthiasis and Nutritional status in Infants and pre-school aged Children in Communities along Ogun River in Abeokuta North Local Government Ogun state**

Community _______________Lat. _________ Long. ________ Altitude___________

**General information of parent/caregiver** Age ______Sex______ Occupation­­­­­­­­­­­­­­­­_____________ **Duration of stay in community**: (a) < 5 years (b) 5-10 years (c) >10 years **Education**: (1) Primary (2) Secondary (3) Tertiary (4) No formal education

**Do you have a generating set**? (a) Yes (b) No

**KNOWLEDGE ABOUT URINARY SCHISTOSOMIASIS AND ITS TRANMISSION**

1. How many pre-school children do you have? __________

| S/No | Identification | Sex | Age (DOB) | Haematuria | Proteinuria | Dirty fingernails? | Trimmed fingernails? |
| --- | --- | --- | --- | --- | --- | --- | --- |
| a. |  |  |  |  |  |  |  |
| b. |  |  |  |  |  |  |  |
| c. |  |  |  |  |  |  |  |

2. Have you heard about the word “urinary schistosomiasis”? (a) Yes (b)No

3. Has any of your wards/children has schistosomiasis before? (a) Yes (b)No

4. a). How do you know that your child or ward has urinary schistosomiasis? ______________

b). what are the symptoms?_________________

5. Have any of your children ever gone/exposed to Ogun River? (a)Yes (b) No (c) Don’t know

6. If yes, which of them? (a) Preschool aged (b) School aged

7. When was the first exposure? (a) At birth (b) before first year (c) 2-6 year of life

8. If at birth, how? (a) Baby was taken to stream (b) water from stream used to bath child at home. (c) Other

9. If during first year of life, how? (a) Child taken to stream (b) water from stream used to bath child at home (c) other

10. If 2-6 years of life, how? (a) Child taken to stream by parents (b) water from stream used to bath child (c) child goes to stream by himself

11. What activity exposes your ward to the river water? (a)Bathing (b) Washing (c) Recreational activities (d) fetching of water (e) others

12. What is the frequency of exposure to stream/river water of preschool children? (a) Everyday (b) once a week (c) Once a month

13. What have you done about the infection? (a) Nothing (b) Taken to the hospital (c) Taken to herbalist

14. Do you think that infection is normal? (a) Yes (b) No

15. What do you suggest can be done to effectively control urinary schistosomiasis infection?

16. Does any of your wards passed out blood in their urine in the last 3 weeks? (a) Yes (b) No

17. Does any of your ward experience painful urination? (a) Yes (b) No

18. What is the distance to the nearest health centre from your household? (a) Far (b) Near

**NUTRITIONAL ASSESSMENT**

| S/No | Identification | Sex | Age (DOB) | Height (cm) | Weight (kg) | Mid-upper arm circumference |
| --- | --- | --- | --- | --- | --- | --- |
| a. |  |  |  |  |  |  |
| b. |  |  |  |  |  |  |
| c. |  |  |  |  |  |  |

**Assessment of Household Sanitation Facilities**

1. What kind of toilet facility do you use? (a) Water Closet (b)Pit with slab (c)Open pit latrine (d)Bush (e) River
2. Do you share your toilet? (a) Yes (b)No If yes how many households? ____
3. Is the toilet facility located within the premises? (a) Yes (b)No
4. How far is the toilet facility from the kitchen/room (a) Near (b) Far
5. What is your source of water for domestic use? (a) Tap (b) River Ogun (c) Well (d)other

**SECTION C - Assessment of Habit**

1. Do you have home slippers? (a)Yes (b)No
2. How often does your ward wear slippers/sandals/shoes? (a)Don’t wear (b)Always (c) Seldom
3. How do you clean your ward’s hands after defecation? (a)Water (b) Paper/Leaves (c )Water + soap (d) none
4. Does your ward pick food from the ground? (a) Yes (b)No
5. Does your ward have any record of swimming/wading/baptism in water? (a) Yes (b)No

**For Infants**:

1. How often do you breastfeed your child per day? (a) Thrice (b)4-5 times (c) > 5 times
2. What is the average duration of a single breastfeed? (a)2 minutes (b)5 minutes ( ) > 5 minutes ( )
3. What is your child’s other food supplements? _________________________________
4. What hygiene practice do you observe before breastfeeding your ward?________________
5. Do you wash your hands before you feed your child? (a) Yes (b)No

**For Older pre-school children**

1. Do you wash your ward’s hands before eating? (a) Yes (b)No
   1. If yes, what do you use? (a)Water (b)Water + Soap
2. At what age did you normally stop breast-feeding your child?­­­­­­­­___________
3. Observe if PSAC has dirty fingernails? (a) Yes (b)No
4. Observe if PSAC has trimmed fingernails? (a) Yes (b)No

**SECTION D - Assessment of Health History**

1. Has your ward had any history of stomach pain? Yes ( ) No ( ) Not sure ( )
2. Has your ward had history of diarrhea/dysentry? Yes ( ) No ( ) Not sure ( )
   1. If yes, when was it? (a) this month (b) last month (c) more than 2 months ago
   2. How long did it last? (a) less than one week (b) 1-2 weeks (c) more than a week
3. Has your ever notice your child pass out worms? Yes ( ) No ( ) Not sure ( )
   1. If yes, at what age was the first time?_____________________________________________
4. How often do you deworm your ward? Always ( ) Rarely ( ) Never ( )
   1. Have you deworm your ward this year? (a) Yes (b) No
   2. If yes, how many times? __________________________________
