# Supplementary material for: Schistosomiasis, intestinal helminthiasis and nutritional status among preschool-aged children in sub-urban communities of Abeokuta, Southwest, Nigeria
Source: BMC Res Notes. 2017 Nov 28;10:637. doi: 10.1186/s13104-017-2973-2 (PMC5706406; doi:10.1186/s13104-017-2973-2)
Supplement: Supplementary file 3 — Additional file 3: Figure S2. Flowchart of the study. [file 13104_2017_2973_MOESM3_ESM.docx]

Sample size calculated

N= 220

Consented participants

N= 241

Preschool-aged children with incomplete questionnaires

N=18

Preschool-aged children with completely filled questionnaires

N=223

Preschool-aged children with incomplete sample

N=56

Preschool-aged children with both urine and faecal samples

N=167

Figure S2A: Flowchart showing preschool-aged children that participated in the study
