# Supplementary material for: Schistosomiasis, intestinal helminthiasis and nutritional status among preschool-aged children in sub-urban communities of Abeokuta, Southwest, Nigeria
Source: BMC Res Notes. 2017 Nov 28;10:637. doi: 10.1186/s13104-017-2973-2 (PMC5706406; doi:10.1186/s13104-017-2973-2)
Supplement: Supplementary file 4 — Additional file 4. Demographics, means Z-score of nutritional indicators used, associated risk factor of infection and correlation analysis. [file 13104_2017_2973_MOESM4_ESM.docx]

Table S1A Demographic characteristic of preschool-aged children

|  | Frequency (N=167) | Percentage (%) |
| --- | --- | --- |
| **Sex** |  |  |
| Male | 83 | 49.7 |
| Female | 84 | 50.3 |
| **Age (Months)** |  |  |
| 0-12 | 31 | 18.6 |
| 13-24 | 35 | 21.0 |
|  |  |  |
|  |  |  |
|  |  |  |
|  |  |  |
|  |  |  |
| 25-36 | 27 | 16.2 |
| 37-48 | 23 | 13.8 |
| 49-60 | 22 | 13.2 |
| 61-71 | 29 | 17.4 |
| **Community sample** |  |  |
| Adebowale-Abowaba | 35 | 21.0 |
| Ago-Ika | 52 | 31.1 |
| Ikereku-Idan | 37 | 22.2 |
| Itun-Seriki | 43 | 25.7 |
| Total | 167 | 100.0 |

*Mean age of Pre-school age children = 3.3years*

Table S2A: Mean z-scores of nutritional indicators and specific helminths infection

| **Infection** | **Infection status** | **Mean HAZ (NE=167)** | **Mean WAZ (NE=167)** | **Mean WHZ (NE=138)** | **Mean BAZ (NE=167)** |
| --- | --- | --- | --- | --- | --- |
| *Ascaris lumbricoides* infection | Infected | -1.51±0.20 | -0.88±0.15 | 0.20±0.18 | 0.25±0.16 |
|  | Non-infected | -1.48±0.19 | -0.92±0.18 | 0.08±0.20 | 0.07±0.18 |
|  | p-value | 0.894 | 0.864 | 0.664 | 0.443 |
| Hookworm infection | Infected | -1.65±0.21 | -0.98±0.16 | 0.12±0.19 | 0.25±0.17 |
|  | Non-infected | -1.40±0.17 | -0.85±0.16 | 0.14±0.19 | 0.11±0.17 |
|  | p-value | 0.375 | 0.588 | 0.961 | 0.577 |
| *Trichuris trichiura* infection | Infected | -1.47±0.64 | -1.37±0.42 | -0.61±0.28 | -0.37±0.23 |
|  | Non-infected | -1.50±0.14 | -0.87±0.12 | 0.17±0.15 | 0.19±0.13 |
|  | p-value | 0.964 | 0.335 | 0.034* | 0.05* |
| *Taenia* spp infection | Infected | -1.67±0.18 | -1.02±0.17 | 0.13±0.20 | 0.20±0.17 |
|  | Non-infected | -1.32±0.20 | -0.77±0.16 | 0.14±0.20 | 0.12±0.18 |
|  | p-value | 0.196 | 0.282 | 0.992 | 0.745 |
| *Strongyloides stecoralis* infection | Infected | -0.52±0.98 | -0.09±0.44 | 0.48±0.51 | 0.43±0.42 |
|  | Non-infected | -1.54±0.13 | -0.93±0.12 | 0.12±0.14 | 0.14±0.13 |
|  | p-value | 0.132 | 0.144 | 0.63 | 0.64 |
| *Trichostrongylus* spp infection | Infected | -1.95±0.25 | -1.27±0.20 | -0.54±0.52 | -0.01±0.32 |
|  | Non-infected | -1.48±0.14 | -0.88±0.12 | 0.15±0.14 | 0.17±0.13 |
|  | p-value | 0.129 | 0.127 | 0.404 | 0.773 |
| Schistosomiasis | Infected | -1.55±0.49 | -0.96±0.34 | -0.14±0.56 | 0.02±0.51 |
|  | Non-infected | -1.49±0.14 | -0.90±0.12 | 0.16±0.14 | 0.17±0.13 |
|  | p-value | 0.913 | 0.891 | 0.57 | 0.76 |
| Helminths only | Infected | -1.51±0.32 | -0.90±0.13 | 0.10±0.15 | 0.18±0.13 |
|  | Non-infected | -1.44±0.15 | -0.91±0.28 | 0.24±0.34 | 0.10±0.30 |
|  | p-value | 0.814 | 0.953 | 0.678 | 0.780 |
| Co-infection of schistosomiasis and intestinal helminths | Infected | -2.04±0.41 | -1.08±0.37 | 0.12±0.55 | 0.25±0.52 |
|  | Non-infected | -1.46±0.14 | -0.89±0.12 | 0.13±0.14 | 0.15±0.13 |
|  | p-value | 0.315 | 0.689 | 0.984 | 0.852 |

Table S3A: Prevalence of Parasite and associated risk factors

|  | Ascariasis | Hookworm | Trichuriasis | Taeniasis | Any helminths |
| --- | --- | --- | --- | --- | --- |
| **Type of toilet facility** |  |  |  |  |  |
| Water closet | 18(22.2) | 15(23.8) | 0(0) | 16(19.0) | 26(20.2) |
| Pit with slab | 45(55.6) | 35(55.6) | 6(66.7) | 42(50.0) | 65(50.4) |
| Open pit latrine | 8(9.9) | 5(7.9) | 2(22.2) | 8(9.5) | 14(10.9) |
| Bush | 8(9.9) | 7(11.1) | 0(0) | 16(19.0) | 21(16.3) |
| River | 2(2.5) | 1((1.6) | 1(11.1) | 2(2.4) | 3(2.3) |
| p-value | 0.115 | 0.252 | 0.054 | 0.538 | 0.458 |
| **Main source of water for domestic use** |  |  |  |  |  |
| Tap | 42(51.9) | 34(54.0) | 5(55.6) | 36(42.9) | 60(46.5) |
| River | 10(12.3) | 7(11.1) | 0(0) | 12(14.3) | 17(13.2) |
| Well | 3(3.7) | 2(3.2) | 0(0) | 5(6.0) | 5(3.9) |
| Multiple source | 26(32.1) | 20(31.7) | 4(44.4) | 31(36.9) | 47(36.4) |
| P-value | 0.877 | 0.831 | 0.593 | 0.070 | 0.319 |
| **Preschool aged children with dirty finger (NE=167)** |  |  |  |  |  |
| Yes | 58 (71.6) | 43(68.3) | 6(66.7) | 55(65.5) | 86(66.7) |
| No | 23 (28.4) | 20(31.7) | 3(33.3) | 29(34.5) | 43(33.3) |
| p-value | 0.034 | 0.318 | 0.838 | 0.589 | 0.114) |
| **PSAC with trimmed fingernails (NE=167)** |  |  |  |  |  |
| Trimmed | 50(61.7) | 41(65.1) | 7(77.8) | 43(51.2) | 78(60.5) |
| Not trimmed | 31(38.3) | 22(34.9) | 2(22.2) | 41(48.8) | 51(39.5) |
| p-value | 0.532 | 0.235 | 0.246 | 0.032 | 0.566 |
| **PSAC washing of hands before eating** (NE=95) |  |  |  |  |  |
| Yes, with water only | 38(46.9) | 26(41.3) | 2(22.2) | 42(50.0) | 57(44.2) |
| Yes, with water and soap | 7(8.6) | 9(14.3) | 1(11.1) | 9(10.7) | 15(10.1) |
| No | 36(44.4) | 28(44.4) | 6(66.7) | 33(39.3) | 59(45.7) |
| p-value | 0.438 | 0.163 | 0.466 | 0.038 | 0.251 |
| **PSAC picking food/ objects from the ground** |  |  |  |  |  |
| Yes | 27(33.3) | 23(36.5) | 2(22.2) | 29(34.5) | 46(35.7) |
| No | 54(66.7) | 40(63.5) | 7(77.8) | 55(65.5) | 83(64.3) |
| Total | 81(100) | 63(100) | 9(100) | 84(100) | 129(100) |
| p-value | 0.667 | 0.303 | 0.528 | 0.436 | 0.045 |

Table S4A: Pearson-product moment correlation of intensity of parasite infection and nutritional indicators

|  | **Pearson r** | **n** | **p-value** |
| --- | --- | --- | --- |
| Nutritional Indicator | ***A. lumbricoides*** | | |
| Wasting (WHZ) | -0.062 | 138 | 0.622 |
|  | **Hookworm** | | |
| Stunting (HAZ ) | -0.024 | 63 | 0.853 |
|  | ***Taenia* spp** | | |
| Underweight (WAZ ) | -0.218 | 84 | 0.046* |
| Wasting (WHZ) | -0.120 | 66 | 0.335 |
| Stunting (HAZ ) | -0.157 | 84 | 0.154 |
| Thinnes (BAZ) | -0.085 | 84 | 0.441 |
|  | ***Strongyloides stecoralis*** | | |
| WAZ | -0.061 | 7 | 0.896 |
| Stunting (HAZ ) | -0.045 | 7 | 0.124 |
|  | *Trichostrongylus* spp | | |
| Wasting (WHZ) | **-0.965** | 4 | 0.035* |
| BAZ | -0.108 | 7 | 0.817 |
|  | ***S. haematobium* ova** | | |
| Stunting (HAZ ) | -0.638 | 6 | 0.173 |
| Underweight (WAZ ) | -0.754 | 6 | 0.083 |
| Wasting (WHZ) | -0.149 | 6 | 0.778 |
| Thinnes (BAZ) | -0.056 | 6 | 0.917 |
|  | ***S. mansoni* ova** | | |
| WAZ | -0.109 | 8 | 0.785 |
| Wasting (WHZ) | -0.033 | 7 | 0.944 |
| Stunting (HAZ ) | -0.116 | 8 | 0.785 |
